# Systematic review of pineal cysts surgery in pediatric patients

**DOI:** 10.1007/s00381-020-04792-3

**Published:** 2020-07-20

**Authors:** Joham Choque-Velasquez, Roberto Colasanti, Szymon Baluszek, Julio Resendiz-Nieves, Sajjad Muhammad, Christopher Ludtka, Juha Hernesniemi

**Affiliations:** 1 Department of Neurosurgery, University of Helsinki and Helsinki University Hospital Helsinki, Helsinki, Finland; 2 Juha Hernesniemi International Center for Neurosurgery, Henan Provincial People’s Hospital, Zhengzhou, China; 3 Department of Neurosurgery, Umberto I General Hospital, Università Politecnica delle Marche, Ancona, Italy; 4 Department of Neurosurgery, Ospedali Riuniti Marche Nord, Pesaro, Italy; 5 Laboratory of Molecular Neurobiology, Nencki Institute of Experimental Biology, Warsaw, Poland; 6 Clinical Department of Neurosurgery, Central Clinical Hospital Ministry of Interior, Warsaw, Poland; 7 Department of Neurosurgery, University Hospital Düsseldorf, Düsseldorf, Germany; 8 Department of Biomedical Engineering, University of Florida, Florida, USA

**Keywords:** Microneurosurgery, Pineal cysts, Sitting position, Supracerebellar infratentorial approach

## Abstract

**Introduction:**

We present a consecutive case series and a systematic review of surgically treated pediatric PCs. We hypothesized that the symptomatic PC is a progressive disease with hydrocephalus at its last stage. We also propose that PC microsurgery is associated with better postoperative outcomes compared to other treatments.

**Methods:**

The systematic review was conducted in PubMed and Scopus. No clinical study on pediatric PC patients was available. We performed a comprehensive evaluation of the available individual patient data of 43 (22 case reports and 21 observational series) articles.

**Results:**

The review included 109 patients (72% females). Ten-year-old or younger patients harbored smaller PC sizes compared to older patients (*p* < 0.01). The pediatric PCs operated on appeared to represent a progressive disease, which started with unspecific symptoms with a mean cyst diameter of 14.5 mm, and progressed to visual impairment with a mean cyst diameter of 17.8 mm, and hydrocephalus with a mean cyst diameter of 23.5 mm in the final stages of disease (*p* < 0.001). Additionally, 96% of patients saw an improvement in their symptoms or became asymptomatic after surgery. PC microsurgery linked with superior gross total resection compared to endoscopic and stereotactic procedures (*p* < 0.001).

**Conclusions:**

Surgically treated pediatric PCs appear to behave as a progressive disease, which starts with cyst diameters of approximately 15 mm and develops with acute or progressive hydrocephalus at the final stage. PC microneurosurgery appears to be associated with a more complete surgical resection compared to other procedures.

**Electronic supplementary material:**

The online version of this article (10.1007/s00381-020-04792-3) contains supplementary material, which is available to authorized users.

## Introduction

The prevalence of benign pineal cysts (PCs) ranges between 0.6 and 23% in the general population [1–7], and is as high as 40% in autoptic series [8]. This large range in reported prevalence is explained by the different types of MRI machine used for the respective studies, the different methods used in defining PC size, and the various types of design and population studies [1–7, 9]. One large study on children and young people in particular showed a PC prevalence of 2% in people under 25 years of age [2]. PCs in the general population are mostly considered normal anatomical variations and the parameters to define pathological PCs in need of treatment are currently unestablished [10–21].

Pediatric patients harbor different physiological, anesthesiological, and neurosurgical features as compared to adult patients. As such, surgically treated pediatric PCs may represent a different and unique entity in clinical practice, which has not previously been properly investigated. Only a few large series on surgically treated PCs have been published in recent years [10–22], and no clinical study on surgically treated PCs in pediatric patients has been reported thus far. In 2013, a review on surgically treated PCs in children was performed. However, the study included only 30 patients collected from small case series [23].

Details on the natural history, clinical features, and surgical outcomes of pediatric pineal cysts are unknown. We present a consecutive case series and a systematic review of surgically treated pediatric PCs. We hypothesized that the symptomatic PC is a progressive disease with hydrocephalus at its final stage. We also propose that PC microsurgery is associated with better postoperative outcomes compared to other treatments in pediatric patients.

## Material and methods

### Population study and design

We report a summary of the retrospective evaluation of the histologically confirmed pediatric PC patients operated on in the Department of Neurosurgery, Helsinki University Hospital between 1997 and 2015. The research methodology used for this purpose was previously presented [22]. The systematic review of all surgically treated pediatric PC patients followed PRISMA guidelines. The American Academy of Pediatrics stablished the upper age limit for pediatric patients as 21 years, strongly discouraging the use of arbitrarily defined age limits [24, 25].

### Data analysis

**Fig. 1:**
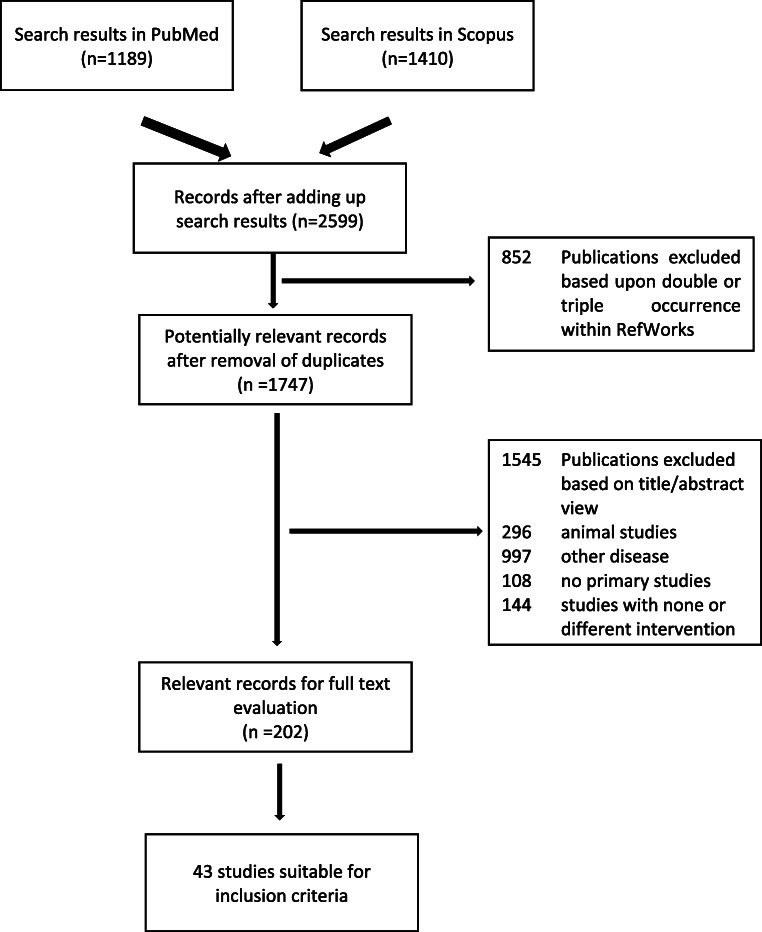
Comprehensive search of the literature and study selection for the surgically treated pineal cysts in pediatric population

In 2016, we registered the “Systematic review and meta-analysis of the clinical outcome following different surgical modalities for the treatment of benign pineal cysts” in PROSPERO (registration number, CRD42016048317). Here, based on the literature search for that review, we conducted a systematic review of all surgically treated pineal cysts in patients with ages ranging from 0 to 21 years old. The literature search was conducted using PubMed (search: *pineal cyst OR glial cyst OR pineal cysts OR pineal gland cyst*) and Scopus [search: *(pineal cyst) OR (glial cyst) OR (pineal cysts) OR (pineal gland cyst)*] databases in November 2019 (Fig. 1). All available original languages were included in the review. Three independent authors performed the comprehensive search of the literature and the data extraction for the study characteristics table. Discrepancies were solved by consensus. Based on a preliminary evaluation of the literature, it was evident that the value of the meta-analysis could be limited due to the wide range of different outcomes that were documented in the few published studies. Subsequently, we provided a narrative synthesis of the findings from the included studies. Since no clinical study on operated pediatric PCs was available, we performed an individual data analysis of all reported patients. A plan for the use of quality assessment tools was outlined beforehand.

### Statistical methods

We performed a descriptive analysis of the study population. For the study of the natural history of the disease, the preoperative presentation of the PCs was categorized into the following groups in correspondence with the PCs’ diameters as proposed in our previous publication [22]: (1) PC patients with hydrocephalus; (2) PC patients with visual symptoms; (3) PC patients with disabling symptoms unrelated with hydrocephalus and tectum compression. The difference between the major PC diameters of the groups was analyzed by the non-parametric Kruskal-Wallis test, and the difference of the PC diameters between each group by the Mann-Whitney *U* test. Besides the individual data analysis, we compared the postoperative outcomes in terms of the extent of surgical resection, immediate neurological complications among the different surgical modalities: (a) microsurgery, (b) endoscopic approach, (c) stereotactic procedures. For this, the chi-square test was used. We avoid comparing the final clinical status among the surgical modalities for the existing risk of selection bias.

R studio was used for the statistical analysis. Only available data was analyzed, and missing data was not extrapolated. *p* value was set at 0.05 for significance.

### Bibliography details

Pediatric patients were retrieved from 43 studies: 22 case reports and 21 retrospective observational series of cases or small cohorts, published between 1947 and 2019 (attached as a supplementary material). Individual participant data was collected to organize the study characteristics table. Original languages of publications included Czech, German, and Japanese for one article each, Spanish in two publications, and English for the remainder [14, 15, 20–23, 26–62].

Of 37 non-selective surgical series with four or more histologically confirmed pineal cysts, no single clinical study focused on the surgical management of PCs in a pediatric population. Some large series have mixed pediatric and adult cases without selectively evaluating the two subgroups.

## Results

### Patient-related variables

We report 109 pediatric patients who were operated on (73% female) with an average age of 14 ± 5.6 years (range 0.25–21). A summary of the findings is provided in Table 1. Five patients had unavailable information about their specific clinical presentation. Eighty-eight percent of the patients reported headache; 39% visual compromise such as blurred vision, diplopia, ptosis, disorders on the eye movements, and Parinaud’s syndrome; 32% nausea; and 25% vomiting. Various other symptoms were reported in 42% of patients: 6% reported syncope, 4% ataxia, 4% impaired concentration, 4% sensory disorders, 4% vertigo, 3% sleep disorders, 3% malaise, 3% weakness, 2% memory deficit, 2% dizziness, 2% dysarthria, 2% tremor, 1% photophobia, 1% facial weakness, 1% hearing impairment, 1% lethargy, 1% hyperprolactinemia, 1% panic attacks, 1% psychological depression, and 1% gait impairment. Two patients were incidental findings after MRI imaging for scoliosis and eye scleritis. Other uncommon manifestations included seizures in 3% of the patients, psychomotor retardation in 2%, trigeminal sensitivity disorder in 1%, precocious puberty in 1%, and West syndrome in 1% of the study population.

**Table 1:** Characteristics of the surgically treated pediatric pineal cysts published in PubMed and Scopus between 1947 and 2019

	All *N*, 109	≤ 10 year old *N*, 27	> 10 year old *N*, 82	*p* value	*N*
Mean age (years)	14 ± 5.6 (0.25–21)	5.8 ± 3.2 (0.25–10)	16.7 ± 3 (11–21)	.	109
Sex: females	79 (73.8%)	16 (59.3%)	63 (78.8%)	0.074	107
Hydrocephalus	37 (35.6%)	7 (25.9%)	30 (39%)	0.252	104
Tectum compression	41 (39.1%)	7 (25.9%)	34 (43.6%)	0.116	105
PC growth in the FU	12 (11%)	2 (7.4%)	10 (12.2%)	0.726	109
PC with unspecific symptoms	42 (39.3%)	15 (55.6%)	27 (33.8%)	0.067	107
PC diameter in mm.	18.6 ± 7.7 (6–40)	14.9 ± 6.3 (6–28)	19.8 ± 7.8 (9–40)	0.006	84
Surgical approach				0.141	99
SCIT	70 (70.7%)	16 (66.7%)	54 (72%)		
OTT	10 (10.1%)	3 (12.5%)	7 (9.3%)		
Transventricular approach	2 (2%)	2 (8.3%)	0		
Endoscopic procedure	11 (11.1%)	2 (8.3%)	9 (12%)		
Stereotactic procedure	6 (6.1%)	1 (4.2%)	5 (6.7%)		
Extent of resection				0.292	92
GTR	72 (78.3%)	16 (76.2%)	56 (78.9%)		
STR	8 (8.7%)	4 (19.1%)	4 (5.6%)		
PR	2 (2.2%)	0	2 (2.8%)		
Fenestration/biopsy	9 (9.8%)	1 (4.8%)	8 (11.3%)		
Third ventriculostomy	1 (1.1%)	0	1 (1.4%)		
Postoperative impairment				0.448	101
None	86 (85.2%)	22 (95.7%)	64 (82.1%)		
Transient visual complications	13 (12.9%)	1 (4.4%)	12 (15.4%)		
Long-lasting impairment	1 (1%)	0	1 (1.3%)		
Dead	1 (1%)	0	1 (1.3%)		
Minimal follow-up in months	16.8 ± 26 (0–122)	13.6 ± 12.5 (0.25–42)	17.6 ± 28.2 (0–122)	0.6	96
Final status				0.291	101
Asymptomatic	81 (80.2%)	18 (78.3%)	63 (80.8%)		
Improvement	16 (15.8%)	3 (13%)	13 (16.7%)		
No improvement	3 (3%)	2 (8.7%)	1 (1.3%)		
Dead	1 (1%)	0	1 (1.3%)		

*GTR*, gross total resection; *FU*, follow-up; *OTT*, occipital transtentorial approach (3 patients reported as suboccipital approach); *PC*, pineal cyst; *PR*, partial resection; *SCIT*, supracerebellar infratentorial approach; *STR*, subtotal resection

In regard to the surgical indications for PCs, 36% of patients had hydrocephalus, 39% presented visual symptoms, and 39% suffered from disabling symptoms unrelated with hydrocephalus and tectum compression. Moreover, the dimensions of the PC increased during the follow-up in 12 patients (11%) and a solid appearance was reported in 10% of the PCs. However, many studies did not report the MRI descriptions of the PCs in their cases. Details on the management of 19 pediatric PC patients operated on at the Helsinki University Hospital are presented in Table 2.

**Table 2:** Characteristics of 19 pediatric PC patients in Helsinki University Hospital. For ordinal data: mean ± SD (min–max)

Age, females (%)	14.6 ± 4.9 (4–20), 17 (90%)
Clinical presentation	Incidental finding (1), headache (15), visual and oculomotor disfunctions (5), nausea and vomiting (6), psychiatric symptoms (2), sensory disorders (2), memory problem (1)
Preoperative mRS (patients)	0 (1), 1 (2), 2 (11), 3 (4), nai (1)
PC size in mm	Length 19.6 ± 5.5 (13–33.5); high 12.5 ± 3.9 (6–23); wide 15.7 ± 3.7 (9–21)
Surgical criteria	Solid tumor suspicion (8), hydrocephalus (7), cyst growth (5), visual disfunction by tectum compression (5), large cysts with minor symptoms (3), suspected fluctuant hydrocephalus (1)
Surgical treatment	Complete microsurgical resection by the supracerebellar infratentorial approach in all cases
Postoperative complications for medical treatment (events)	CSF leak (3), bacterial meningitis (3), small minimally symptomatic bleeding in the operative site (1)
Immediate mRS (patients)	0 (8), 1 (8), 2 (2), 3 (1)
Long-term mRS (patients)	0 (19)
CCOS total (patients)	15 (7), 16 (12)

*CCOS*, Chicago Chiari outcome scale; *mRS*, modified Rankin scale; *MRI*, magnetic resonance imaging; *nai*, non-available information; *PC*, pineal cyst; *SD*, standard deviation

### Disease characteristics

**Fig. 2:**
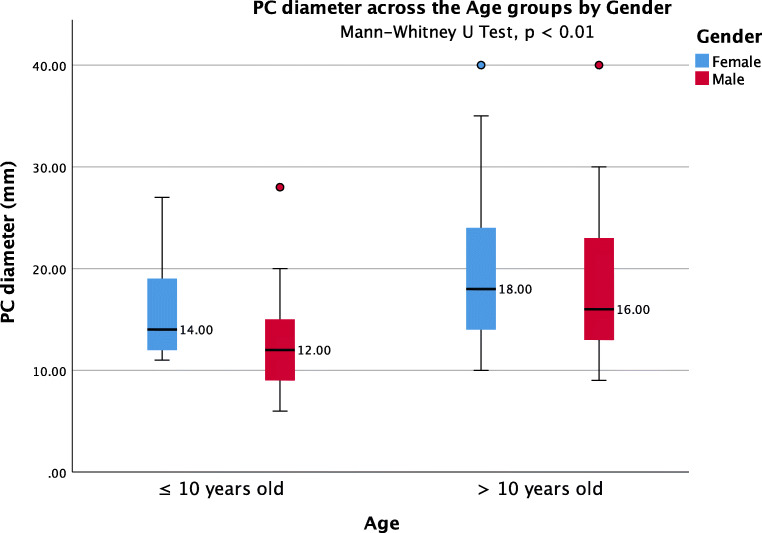
Pineal cysts diameters across the age groups controlled by gender

**Fig. 3:**
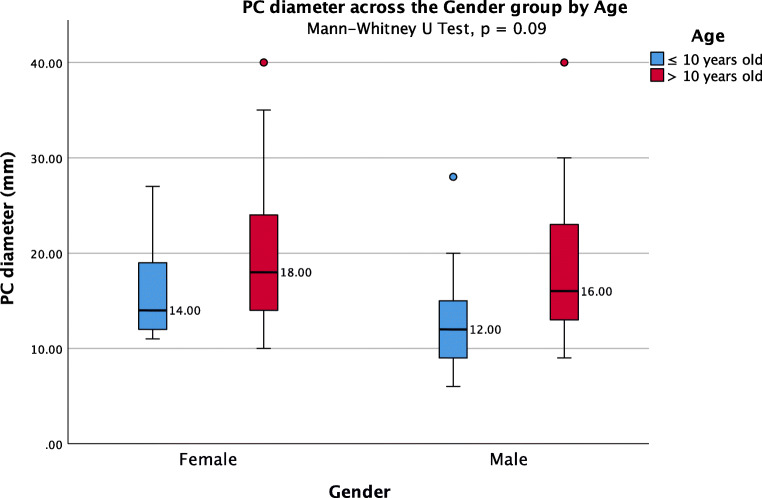
Pineal cysts diameters across the gender groups controlled by age

**Fig. 4:**
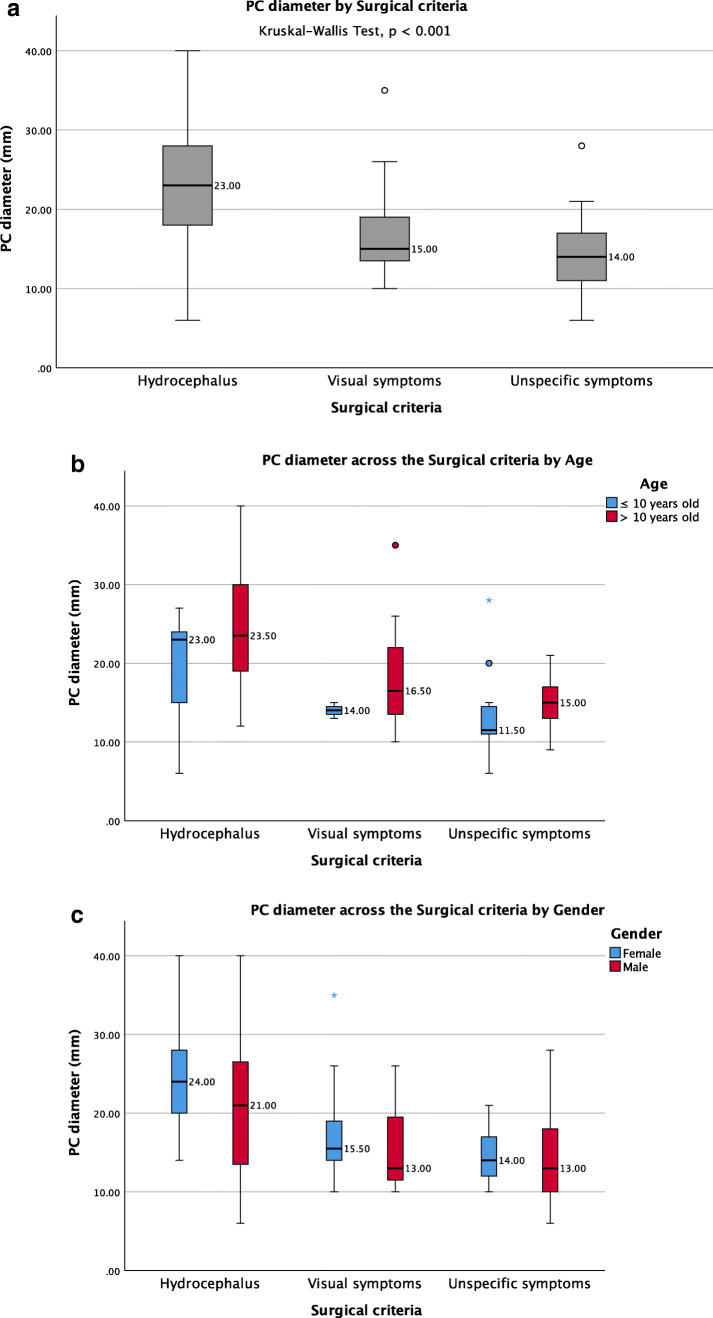
Pineal cysts diameters across the surgical criteria groups (**a**), controlled by age (**b**), and controlled by gender (**c**)

The average PC diameter was 18.6 ± 7.7 mm (range 6–40). In 16 patients, the cyst size was not reported, and in 9 patients, the reported size was nonspecific (smaller than 20 mm in three, between 10 and 35 mm in four, and larger than 20 mm in two). The average PC size in 10-year-old or younger patients [14.9 ± 6.3 (6–28) mm] was smaller compared to patients older than 10 years [19.8 ± 7.8 (9–40) mm] (Mann-Whitney *U* test, *p* < 0.01) (Fig. 2). The PC size did not differ statistically between males and females (Fig. 3). The analysis on the reported PC sizes and preoperative clinical presentation of the patients resulted in the following findings: (1) the average diameter of PCs associated with hydrocephalus was 23.5 ± 8.1 mm (range 6–40); (2) the average diameter of PCs associated with visual symptoms was 21 ± 7.7 mm (range 10–40); (3) the average diameter of PCs associated with visual symptoms without hydrocephalus was 17.4 ± 6.3 mm (range 10–35); (4) the average diameter of all PCs unrelated to hydrocephalus and tectum compression was 14.5 ± 4.3 mm (range 6–28); (5) the difference in PC dimensions between these subgroups of PC patients was statistically significant (Kruskal-Wallis, *p* < 0.001) (Table 3) (Fig. 4). Moreover, under paired analysis PCs with hydrocephalus had statistically different cyst sizes compared to PCs with tectum compression without hydrocephalus (Mann-Whitney *U* test, *p* < 0.01). The PC size of patients with psychomotor retardation, West syndrome, and one incidental finding were mostly unavailable. The other incidentally found PC increased in size during the follow-up from 20 to 24 mm.

**Table 3:** Pineal cyst (larger) diameters on the subgroups of surgically treated pediatric patients

	Mean ± SD (min-max) mm	*N*
PC with hydrocephalus	23.5 ± 8.1 (6–40)	29
PC with visual symptoms	21 ± 7.7 (10–40)	31
PC with visual symptoms without hydrocephalus	17.4 ± 6.3 (10–35)	19
PC enlargement during FU, last preoperative measurement	17.6 ± 4 (13–24)	10
PC enlargement during FU, initial preoperative measurement	15 ± 5.9 (6–27)	10
PC with unspecific symptoms	14.5 ± 4.3 (6–28)	34
All PC	18.6 ± 7.7 (6–40)	82

*FU*, follow-up; *PC*, pineal cysts, *SD*, standard deviation

Within the study population, 14% (11 PCs) of females and 4% (1 PC) of males harbored cysts that increased in size during the follow-up period (*p* > 0.05) [23, 40, 41, 52, 53, 56, 58]. Additionally, most of these patients were 10 years old or older (*p* > 0.05). The average PC diameters were 15 ± 5.9 mm (range 6–27) in the initial preoperative MRI and 17.6 ± 3.9 mm (range 13–24) at the last preoperative evaluation. The average time between the initial observation and the date of surgery in this group of patients was 3.3 ± 2.8 years (range 0–7). The shortest recorded time of PC growth was over 5 days following re-apoplexy. The reasons for progressive PC growth remained unknown for the rest of the patients.

### Imaging

More than 50% of the patients did not report imaging studies. The detailed information on the reported studies widely vary and did not reveal any typical PC aspect on the imaging except for its rounded and well-defined borders. The few CT scan reports described calcified rings, nodular calcifications, fluid levels, acute intracystic hemorrhages, and ring enhancements following contrast administration [21, 36, 38, 40, 43, 46, 48]. Sixty-one (56%) patients did not have reports on the T1WI MRI sequences. Twenty PCs harbored some degree of low, moderate, and high hyperintense signal compared to the CSF, while 9 PCs were isointense to the CSF. After contrast delivery, 22 patients showed ring enhancement, 11 patients showed diffuse enhancement of a solid lesion, and seven PCs included intracystic septa [23, 26, 28, 30–32, 34, 35, 38, 40–43, 45–48, 50, 52, 53, 56–59, 62]. On the other hand, 74 patients did not report T2WI MRI sequences. On the reported cases, the PC content was isointense to the CSF in 25 patients, hypointense in three patients, and hyperintense in other three patients. Intracystic fluid levels were reported in seven patients and acute hemorrhages in five others [23, 26, 28, 30, 32, 35, 38, 40, 44, 46, 48, 52, 53, 62].

Patients with PC that increased in size during the follow-up demonstrated different features [23, 40, 41, 52, 53, 56, 58]. Few of them followed apoplectic events with signals of intracystic hemorrhages. Others remained isointense to the CSF. Some PCs included intracystic septa, ring enhancements, or even diffuse enhancement of solid components. Thus, the mechanisms behind the cyst growth in pediatric patients seem to vary between the cases. Apoplectic events, osmotic and mechanic pressure processes, and hormonal mechanisms among others would be involved and further research should be focused on this topic.

### Pathological findings

All the reported cases except one had a histological confirmation of a benign PC. The pathological study was absent in one patient since the PC shrunk after endoscopic third ventriculostomy [30]. The published reports only detailed histopathological findings in 19 patients [31, 36, 43, 45–50, 52, 54–57]. Three well-defined layers without atypical cells, an inner glial layer, a middle pineal cell layer, and an external fibrous capsule, were mostly reported. Few reports only included an inner glial and an external pineal cells layer. Rosenthal fibers, ependymal cells lining the inner glial layer, and deposits of calcification within the pineal layer were also reported in few cases. Finally, apoplectic PCs included granular bodies with hemosiderin-containing macrophages in the inner layers. The PC content was reported as a xanthochromic fluid with high concentration of protein material, exfoliated pinealocytes, and lymphocytes. Hemosiderin-landen macrophages and erythrocytes were reported in apoplectic PCs. Immunohistochemical studies revealed strong immunoreactivity for synaptophysin in the pineal cells layer and strong reactivity for GFAP in the glial layers. A neurofilament immunostaining was present around intralobular vessels as well, while EMA and Ki-67 reactivity were negative. In our series, all PCs were reported as benign glial cysts by an experienced pathologist. As a reference, chromogranin stain positive in pineal parenchymal tumors remains negative for PCs [22, 63].

### Surgical intervention

Information regarding the surgical approach and the extent of surgical resection is detailed in Table 4. PC microneurosurgery was the most common surgical procedure and correlated with better rates of complete resection (chi-square test, *p* < 0.001). Among the different microsurgical approaches, none was statistically superior in terms of surgical resection. The endoscopic procedures included third ventriculostomy alone, cyst fenestration, partial resection, and complete resection. Complete endoscopic PC resection was performed in four patients. However, two of them required a microsurgical technique (endoscopic assisted approaches).

**Table 4:** Postoperative clinical and radiological status across the surgical procedure groups

	Endoscopic approach	Microsurgery	Stereotactic procedure	*p* value
	*N*, 10	*N*, 75	*N*, 6	
Extent of resection				< 0.001
Complete resection	4 (40%)	67 (89%)	0	.
Incomplete resection	2 (20%)	8 (11%)	0	
Fenestration	3 (30%)	0	6 (100%)	.
Ventriculostomy	1 (10%)	0	0	
Immediate neurological complications				0.14
None	9 (90%)	69 (92%)	5 (83%)	.
Transient visual Impairment	1 (10%)	12 (16%)	0	.
Worse diplopia	0	1 (1%)	0	.
Death	0	0	1 (17%)	.
Preoperative symptoms at the last evaluation				NP
Improved	0	15 (20%)	0	.
No complaints	10 (100%)	65 (87%)	4 (67%)	.
No improvement	0	2 (3%)	1 (17%)	.
Death	0	0	1 (17%)	.

*NP*, not performed

PC size among the various subgroups of treatment was statistically similar. However, in regard to the date of treatment, the stereotactic procedures were performed between 1947 and 1993, and the transventricular approaches were performed in 1987. Further, the occipital transtentorial approaches were reported as late as 2012, the endoscopic approaches between 1995 and 2016, and the infratentorial supracerebellar approach as late as 2019.

### Surgical findings

Very few studies reported macroscopic details of surgically treated pediatric PCs [38, 46, 50, 52, 54–56, 59, 61]. A soft, grayish, rounded, and well-defined cyst with or without a vascularized wall was commonly described. This encapsulated cyst with a thin membrane and dense adhesions to the tectal plate works as a “ball-valve” fashion over the aqueduct and contains a clear xanthochromic fluid [61]. We edited a supplementary video of a surgically treated pediatric PCs under sitting position and a paramedian supracerebellar infratentorial approach (VIDEO 1).

### Follow-up and outcome

In the immediate postoperative neurological evaluation, 85% of patients did not demonstrate any neurological complication. However, 14% of patients who underwent the SCIT approach developed transient visual impairment and, in one additional patient (1.4%), diplopia worsened temporarily after surgery. One (10%) patient in the endoscopic group had a transient Parianud’s syndrome. Two (20%) patients who underwent the occipital transtentorial approach had a transient visual compromise as well. The first published stereotactic procedure for a PC reported patient death during surgery in 1947. None of the transventricular approaches reported neurological complications. No statistical difference in the immediate postoperative neurological impairment was found among the different treatment modalities (Table 4).

The period of follow-up was unreported in 12% of patients, while 23% of patients had less than or equal to 3 months of follow-up and 65% of patients had more than 3 months of follow-up at least. The average minimum follow-up across patients was 16.8 ± 25.7 months (range 0–122). At the last follow-up and excluding the noted deceased patient, 80% of patients did not report any complaints, 16% had notable improvement of their primary symptoms, and three (3%) patients did not have any improvement of their major symptoms. At the time of the last evaluation, no statistical difference in clinical status was observed between patients regarding the various extent of surgical resection, nor among the different surgical procedures.

At the long-term follow-up, symptoms and signs that improved after surgical treatment included headache, nausea, vomiting, unspecific visual compromise, diplopia, blurred vision, gaze paresis, ptosis, strabismus, Parinaud’s syndrome, tremor, hyperprolactinemia, precocious puberty, impaired concentration, ataxia, dizziness, malaise, vertigo, fatigue, seizures, panic attacks, syncope, paresthesia, memory deficit, weakness, psychological depression, lethargy, delayed speech development, and dysarthria. The West syndrome case, the psychomotor retardation case, and a Parinaud’s syndrome case did not demonstrate change at the time of their long-term evaluation. Sleep disorder, memory deficit, inability to converge the eyes, and some cases of headache had mixed recovery with good outcomes in some patients and unchanged status in others.

## Discussion

In regard to the natural evolution of the disease, the aforementioned findings are similar to those we reported previously in the largest series of surgically treated pineal cysts [22]. Thus, we propose that surgically treated PCs represent a progressive disease with acute or progressive hydrocephalus at its final stage. Furthermore, we suggest that young females with active sexual hormone status (> 10-year-old) would be the pediatric group at highest risk for disease progression, and recommend that further research should focus on this matter. In regard to the possibility of PCs in female patients increasing in size, hormonal implications could play an important role in the natural history of this disease, as suggested by previous publications [36, 64].

Contrary to our findings, a large epidemiological study on people under 25 years old conducted by Al-Holou et al. concluded that the prevalence of pineal cysts that increase in size during the follow-up was very low. Most of the PCs remained stable or decreased in size along a mean follow-up of 3.4 years. However, that study was designed to evaluate PCs of 5 mm or larger, with average PC diameters of 9.7 ± 3.8 mm at initial diagnosis [3]. A comprehensive analysis of these findings may conclude that most small PCs remain stable during the follow-up period. However, PCs with diameters larger than 15 mm might represent a pathological entity and require treatment. Further studies on different age groups should be performed to draw appropriate conclusions.

In regard to the surgical procedures and their postoperative outcomes, some information was obtained despite being limited by the unavailability of clinical studies on surgically treated PCs. Individual data analysis offers limited usefulness regarding statistical analysis, as the selection bias of the patients is undefined. Regardless, some of the very consistent conclusions regarding patient outcome include (a) 96% of pediatric patients improved their symptoms or became asymptomatic after the surgical treatment of their PCs and (b) PC microneurosurgery was associated with a better extent of resection yet with similar postoperative clinical status compared to other surgical procedures.

The endoscopic procedures demonstrated symptom-free status in all patients at their last evaluation, although an accurate analysis of the data showed that all the cases followed a careful preoperative selection. Indications for endoscopic procedures were restricted to patients with symptoms related to hydrocephalus or visual disturbances, and no patient with unspecific symptoms was included in this group of treatment. The patient who underwent third ventriculostomy alone showed a progressive reduction of the PC over the 36 months of follow-up. Of the six patients who underwent stereotactic procedures, four became asymptomatic, one patient died, and one patient with psychomotor retardation remained unchanged. Moreover, a PC recurred at 71 months, presenting with a larger size and associated hydrocephalus, and required re-aspiration [55]. These results suggest a reduced safety and effectiveness of the stereotactic procedure for the management of pediatric PCs. In the microsurgical group of treatment, only three of the 10 patients who underwent an occipital transtentorial approach were asymptomatic at the last evaluation. Moreover, four patients did not recover the ability to converge their eyes 2 years after surgery. In comparison, 60 of the 70 patients who underwent the SCIT approach were asymptomatic, and nine additional patients showed an improvement of most of their symptoms at the last evaluation. One of them did not show improvement of the preoperative Parinaud’s syndrome. Another patient with a psychomotor retardation operated in 1987 had an unchanged outcome [60]. New deficits such as memory, concentration, and sleep disorders were also reported in three patients.

The systematic review performed strongly supports our previous findings on the progressive character of surgically treated PCs. The current surgical management of pediatric PCs seems safe. However, residual PCs are frequent after endoscopic or stereotactic procedures in contrast to the minor rate of remnants after microneurosurgery, particularly following the SCIT approach. Failure to achieve clinical improvement of surgically treated PC patients should be avoided by proper surgical selection of patients, focusing on the disease-related clinical presentation. As previously mentioned, the main limitation of this systematic review is the data being retrieved from small series of cases that did not allow us to draw solid conclusions.

## Conclusion

Surgically treated pediatric PCs appear to be a progressive disease, which starts with unspecific symptoms with mean cyst diameters of 15 mm, and progresses with visual impairment and hydrocephalus at the final stage. PC microneurosurgery seems to be associated with a better extent of surgical resection compared to endoscopic and stereotactic procedures. Failure to achieve clinical improvement of surgically treated PC patients should be avoided by proper surgical selection of patients. Further research is required on this topic.

## Electronic supplementary material

ESM 1 (XLSX 25 kb)

ESM 2 (MP4 45,805 kb)

## Abbreviations

CCOS: Chicago Chiari outcome scale
HUH: Helsinki University Hospital
mRS: modified Rankin scale
MRI: magnetic resonance imaging
PC: pineal cyst
SCIT: supracerebellar infratentorial paramedian approach
